# Multi-Omics Data Integration Clustering for Cancer Subtypes Identification Based on Motif High-Order Similarity Graph and Tensor Regularization

**DOI:** 10.3390/genes17050587

**Published:** 2026-05-21

**Authors:** Hongbin Yan, Fuyan Hu

**Affiliations:** School of Mathematics and Statistics, Wuhan University of Technology, Wuhan 430070, China; 347507@whut.edu.cn

**Keywords:** multi-omics data integration, cancer subtyping, similarity graph, high-order relationship

## Abstract

Background: The precise identification of cancer subtypes through the integration of multi-omics data has emerged as a key research direction in bioinformatics. Among existing multi-omics integration methods, similarity graph-based clustering algorithms have attracted widespread interest owing to their capacity to effectively characterize the association patterns between samples. However, the majority of existing methods primarily focus on first-order relationships among samples while ignoring the prevalent high-order neighborhood relationships, and fail to fully exploit the complementary information from different omics. Methods: To address these limitations, we propose an innovative multi-omics integration framework termed MHSGTR, which integrates multi-omics data by combining Motif high-order similarity graphs and tensor regularization to identify cancer subtypes. Specifically, MHSGTR introduces Motif theory to construct a high-order similarity graph and designs a high-order graph learning term to obtain a hybrid similarity that integrates both first-order and high-order information, thereby capturing the latent high-order structural information among samples. For multi-omics data integration, we employ third-order tensor regularization constraints to explore complementary information across multi-omics data, coupled with an attention module to adaptively learn omics-specific weights for constructing a consensus similarity graph. Final clusters are derived via spectral clustering. Results: Comprehensive experiments on eight TCGA cancer datasets and a case study on adrenocortical carcinoma (ACC) demonstrate that MHSGTR achieves superior clustering performance and identifies cancer subtypes with significant biological differences, showcasing its effectiveness in robust multi-omics integration.

## 1. Introduction

Cancer is a multifactorial disease with striking heterogeneity in terms of molecular mechanisms, pathological features, and clinical manifestations [1]. Within real clinical settings, patients with the same cancer type commonly display substantial differences in treatment response and survival outcomes, even when receiving therapeutic regimens [2]. This clinical phenomenon strongly substantiates the existence of prominent molecular heterogeneity in cancer. With continuous advances in high-throughput sequencing technologies, numerous studies have dissected the complexity and underlying causes of cancer heterogeneity by integrating multi-omics data analyses, providing effective strategies for cancer subtype identification. Accordingly, the efficient fusion of multi-omics data has become one of the key research topics in the field of cancer subtyping in recent years.

Based on distinct data integration strategies, existing integrative methods can be generally divided into three types: early integration, late integration, and intermediate integration [3]. Early integration methods, such as LRAcluster [4], directly concatenate different omics datasets and transform the problem into a single-omics clustering task. However, due to the inherent discrepancies in the distribution and biological functions of different omics data, simple feature concatenation often compromises clustering performance. Late integration methods, including PINS [5] and COCA [6], first perform independent clustering on each omics dataset and then integrate multiple clustering outcomes to generate the final clustering result. Nevertheless, late integration approaches rely solely on precomputed clustering results during the fusion stage, which hinders the full exploitation of latent information within individual omics data and tends to result in the loss of certain critical information. Currently, the majority of multi-omics cancer subtyping methods fall into the category of intermediate integration, such as SNF [7], which aim to capture synergistic and complementary information across different omics layers to achieve more efficient information fusion.

Intermediate integration methods based on similarity graphs have become highly popular in recent years. These methods attempt to integrate similarity information from all omics data to obtain a consistent similarity graph, and then accurately identify cancer subtype structures by combining spectral clustering algorithms. For example, Cancer integration via multikernel learning (CIMLR) [8] employs Gaussian kernel functions to construct 55 kernels for each omics dataset, combines these kernels with low-rank constraints, and then fuses them into a unified similarity graph through adaptive weighting. Within the CIMLR framework, wMKL [9] incorporated prior weights into the construction process of each kernel and learned inter-sample relatedness through integrating multiple kernels generated from different omics, thereby achieving higher subtyping accuracy. However, omics data are typically characterized by high dimensionality, small sample sizes, and high noise levels. Moreover, omics data themselves lack an inherent graph structure, making similarity graphs constructed directly from such data highly susceptible to noise interference. Consequently, recent studies have gradually integrated dimensionality reduction representation learning with similarity graph learning, which not only reduces noise and redundant information but also further optimizes the quality of similarity graphs and the performance of subsequent analyses. For example, MSCLRL [10] improved the quality of each omics similarity matrix by joint latent representation and self-expressive similarity learning, thereby successfully identifying biologically meaningful cancer subtypes. MCLS [11] adopted PCA for feature selection on each omics dataset, and constructed high-quality latent subspaces via singular value decomposition to improve clustering performance.

As deep learning has exhibited remarkable advantages in feature extraction, numerous studies have combined deep learning with similarity graph learning to improve the accuracy of tumor subtype identification. DSML [12] utilized deep neural networks for learning low-dimensional embeddings from each omics dataset, and further improved cancer subtype identification by integrating self-expressive similarity learning on this basis. MoAGL-SA [13] achieved adaptive fusion of multi-omics data by combining similarity graph learning, graph convolutional networks, and self-attention mechanisms, significantly boosting subtype classification performance. DLSF [14] inserted a shared self-expressive module placed between the encoding and decoding branches to alleviate the heterogeneity of multi-omics data and realize adaptive nonlinear fusion of omics information. MOCR [15] constructed similarity graphs via self-expressive learning, modeled both intra-omics features and inter-omics correlation features simultaneously, and achieved efficient learning of multi-omics similarity graphs, thereby significantly enhancing cancer subtype identification.

Although the aforementioned similarity graph-based methods have achieved promising clustering performance in cancer subtype identification, they still suffer from certain limitations. First, most methods rely on first-order similarity to characterize relationships among samples, and such a representation is insufficient to capture the true underlying correlation structure between samples. Especially in omics data with high noise and redundancy, the Laplacian matrix constructed from first-order similarity graphs tends to yield unstable clustering results during spectral clustering. To address these issues, several studies have attempted to explore high-order neighborhood information among samples to further enhance the expressive power of omics-specific similarity graphs. For instance, SFHOP [16] defined high-order proximity relationships between samples and performed similarity fusion, successfully identifying biologically meaningful cancer subtypes. GAHGC [17] revealed the intrinsic structures of samples and features via high-order correlations in hypergraphs, achieving bi-directional co-clustering of samples and features and significantly improving clustering performance. HyperTMO [18] enhanced the representation ability of omics features by constructing hypergraph convolutional networks, thereby improving the accuracy of patient classification. These studies clearly demonstrate that introducing high-order relationships into traditional first-order similarity graphs helps to comprehensively characterize the complex structures among samples, thus significantly improving the accuracy of cancer subtype identification. Second, most methods either assign equal weights to each omics layer or align multiple similarity graphs to a consensus graph. Such strategies fail to fully exploit the complementary information across views, resulting in suboptimal similarity graphs for clustering.

To address these issues, we propose a novel multi-omics clustering method based on Motif high-order similarity graphs, termed MHSGTR. First, MHSGTR constructs both first-order similarity graphs and Motif high-order similarity graphs. By designing a high-order graph learning term, complex high-order connectivity patterns are effectively incorporated into the first-order similarity matrices. Second, to effectively exploit the rich information across different omics layers, third-order tensor regularization and an attention mechanism are adopted to mine complementary information among omics, thereby enhancing the representation ability of the consensus similarity graph. Finally, the rationality and effectiveness of MHSGTR are validated through extensive experiments on eight cancer datasets and a case study of ACC.

## 2. Materials and Methods

### 2.1. Implementation Pipeline of the MHSGTR Algorithm

**Figure 1:**
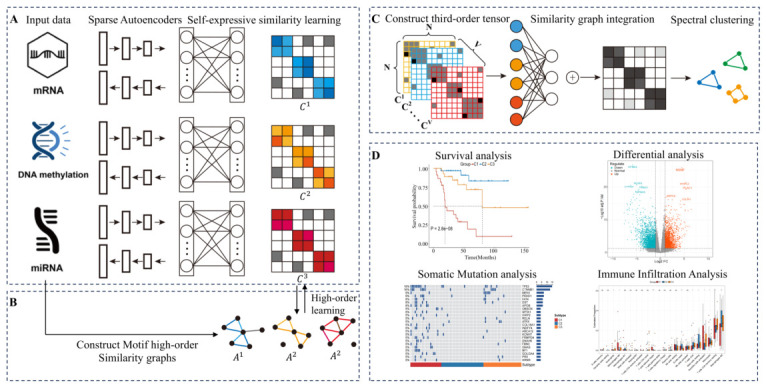
The workflow of MHSGTR algorithm.

The flowchart of the MHSGTR algorithm is depicted in Figure 1 and comprises four main steps: (A) Sparse autoencoders are employed to perform deep dimensionality reduction learning on the raw omics data, and self-expressive subspace learning is then adopted to construct similarity graphs for each omics type. (B) High-order similarity graphs containing Motif-based high-order structural information are constructed from the raw data, and a high-order graph learning term is introduced to effectively integrate complex high-order connectivity patterns into the self-expressive matrices. (C) Weighted fusion of the omics-specific similarity graphs is achieved via third-order tensor-based regularization and an attention mechanism. Based on the constructed consensus similarity graph, spectral clustering is utilized to generate the final clustering results. (D) Downstream statistical analysis is conducted.

**Stage 1: Single-Omics Similarity Learning Based on Sparse Autoencoders and Self-Expressiveness.** Stage 1 focuses on constructing reliable single-omics similarity graphs, which serves as a fundamental basis for subsequent multi-omics integration and clustering. To achieve this, we combine sparse autoencoders (for dimensionality reduction) with self-expressive subspace learning (for similarity graph construction), as motivated by the following insights. Integrating deep neural networks with similarity graph learning has been demonstrated to enhance graph quality by reducing noise and redundancy, thereby improving clustering performance [19,20]. We adopt an autoencoder-based deep learning model for dimensionality reduction. Previous studies [21] have demonstrated that imposing sparsity constraints on the hidden layers of autoencoders can encourage sparse feature distributions, which facilitates feature selection, suppresses noise, and enhances the feature extraction performance of autoencoders. Inspired by this, we introduce an $L_{2,1}$-norm penalty on the weights of the hidden layer to further strengthen the autoencoder’s representational capacity, and its loss function can be expressed as:

(1) $$\min\;\sum_{v=1}^{V}\frac{1}{2n}{∥X^{v}-\tilde{X^{v}}∥}_{F}^{2}\;+\;\alpha {∥W^{\left(1\right)}∥}_{2,1}$$

where $X^{v}$ and $\tilde{X^{v}}$ denote the original data and reconstructed data of the *v*-th omics, respectively. $W^{\left(1\right)}\in R^{d^{v}\times p_{1}}$ denotes the first-layer projection matrix of the autoencoder, where $d^{v}$ represents the number of input features in the *v*-th omics data and $p_{1}$ denotes the dimension of the first hidden representation. Since each row of $W^{\left(1\right)}$ corresponds to an original omics feature, imposing the $L_{2,1}$-norm on $W^{\left(1\right)}$ can induce row-wise sparsity. Consequently, the weights connecting irrelevant or noisy features to all hidden units are jointly shrunk, thereby achieving feature-level selection.

In the self-expressive learning framework, the self-expressive module is implemented as a fully connected layer without activation functions between the encoder and decoder. This module captures the reconstruction relationships among samples in the latent space, with regularization constraints imposed on its parameters. Consequently, the latent representations learned by the encoder are not only used for input reconstruction but also participate in backpropagation together with the self-representation constraints, enabling the network to simultaneously optimize representation learning and similarity structure learning during training. The loss function based on sparse autoencoder and self-expressive learning can be formulated as:

(2) $$\min\;\sum_{v=1}^{V}\frac{1}{2n}{∥X^{v}-\tilde{X^{v}}∥}_{F}^{2}\;+\;\alpha {∥W^{\left(1\right)}∥}_{2,1}+\lambda_{1}{∥Z^{v}-Z^{v}C^{v}∥}_{F}^{2}+\lambda_{2}{∥C^{v}∥}_{F}^{2}$$

where α, $\lambda_{1}$ and $\lambda_{2}$ are hyperparameters, and $C^{v}\in R^{N\times N}$ denotes the similarity graph of the *v*-th omics.

**Figure 2:**
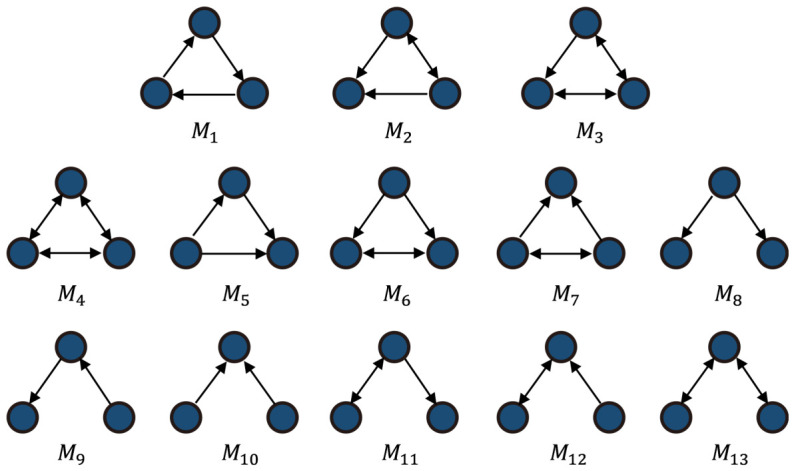
Thirteen forms of triangular Motif.

**Stage 2: Motif-Based High-Order Similarity Mining.** Motif is a local subgraph structure that appears significantly more frequently in complex networks than in random networks, and is regarded as the basic building block for characterizing the local organizational patterns of networks. When two samples are adjacent and form multiple closed triplets with common neighbors, they are more likely to belong to the same cluster. Compared with similarity matrices that rely only on first-order relationships, high-order similarity constructed from motif structures can maintain a more stable neighborhood structure even in the presence of noisy edges, accidental connections, or local sparsity. This helps improve the clarity of clustering boundaries and the stability of clustering results, making the clustering more consistent with the real organizational structure of the network. As illustrated in Figure 2, Benson et al. [22] proposed thirteen types of triangular motifs and demonstrated their importance in social network analysis. Among them, Motif M4 and Motif M13 are suitable for undirected network structures. Based on the above studies, our work selects Motif M4 as the high-order structural unit to construct the motif matrix, thereby better capturing high-order relationships between nodes.

To incorporate motif information into the subsequent modeling process, we adopt the motif matrix construction strategy proposed by Li et al. [23], using the triangular Motif M4 structure to mine high-order topological information and construct the high-order similarity matrix. First, the Gaussian kernel function is applied to the raw omics data to calculate the similarity among individual samples:

(3) $$s_{ij}=\exp\left(-\frac{{∥x_{i}-x_{j}∥}_{2}^{2}}{2\sigma^{2}}\right)$$

Subsequently, a similarity threshold ε is set to the median value of the corresponding omics similarity matrix to improve the robustness of threshold selection. Based on the thresholding result, the binary adjacency matrix $W^{0,1}$ and weighted adjacency matrix *W* are constructed as follows:

(4) $$W_{ij}^{0,1}=\left\{\begin{array}{cc} 1, & s_{ij}\geq \varepsilon \\ 0, & \mathrm{else} \end{array}\right.,\;W_{ij}=\left\{\begin{array}{cc} s_{ij}, & s_{ij}\geq \varepsilon \\ 0, & \mathrm{else} \end{array}\right.$$

On the basis of the binary adjacency matrix $W^{0,1}$, We define the triangle set as $T=\left\{\left\{i,j,k\right\}|W_{ij}^{0,1}=W_{ik}^{0,1}=W_{jk}^{0,1}=1,\;i<j<k\right\}$. The elements of the Motif M4 matrix are then defined as the number of node-level motifs that any two nodes jointly participate in, given by ${\left(W_{M}\right)}_{ij}=\left|\left\{k|\{i,j,k\}\in T\right\}\right|,\;i\neq j$. Then, the Hadamard element-wise product between the Motif M4 matrix $W_{M}$ and the weighted adjacency matrix *W* are conducted to obtain the motif similarity matrix *M* containing high-order structural information. Although the initial similarity graph *S* only characterizes the first-order similarity relationships among samples and inevitably contains noise, it generally contains important information about the topological structure of the graph. To fully exploit the complementary advantages of first-order similarity and Motif-based high-order structures, we linearly fuse the initial similarity graph *S* with the motif similarity matrix *M* to obtain the comprehensive high-order similarity graph:

(5) A=ηS+(1−η)M

where η is a trade-off parameter that adjusts the relative contributions of first-order and high-order information. To achieve mutual learning between the high-order graph *A* and the self-expressive similarity graph *C*, we integrate information from *A* through a regularization term. Accordingly, the loss function for Motif-based high-order similarity learning can be expressed as:

(6) $$\sum_{v=1}^{V}\lambda_{3}{∥A^{v}-C^{v}∥}_{F}^{2}$$

where $\lambda_{3}$ is a hyperparameter. Through the above equation, the self-expressive similarity graph is endowed with high-order information, enabling it to better explore the high-order relationships among samples.

**Stage 3: Similarity graph integration based on tensor regularization and attention mechanism.** After obtaining the similarity graph for each omics, a critical issue is to select an appropriate graph integration strategy to derive a consensus graph. Recent studies have demonstrated that tensor nuclear norm-based methods can effectively explore the interactive relationships among different omics [24,25]. Therefore, we adopt the graph fusion strategy developed by Zheng et al. [25]. Specifically, the similarity graph of each omics is stacked to form a third-order tensor, denoted as $C^{\prime }=\left\{C^{1},C^{2},\ldots ,C^{V}\right\}\in R^{N\times N\times V}$, where $C^{v}\in R^{N\times N}$ is the similarity graph of the *v*-th data. Then, a rotational transformation is applied to the tensor to obtain a new representation, on which a t-SVD-based tensor nuclear norm is introduced to strengthen the model’s capacity in capturing high-order multi-omics relationships. The corresponding loss function is defined as follows:

(7) $$\begin{array}{cc} \; & \min\;\sum_{v=1}^{V}\frac{1}{2n}{∥X^{v}-\tilde{X^{v}}∥}_{F}^{2}+\alpha {∥W^{\left(1\right)}∥}_{2,1}+\lambda_{1}{∥Z^{v}-Z^{v}C^{v}∥}_{F}^{2}+\lambda_{2}{∥C^{v}∥}_{F}^{2}\\ \; & +\lambda_{3}{∥A^{v}-C^{v}∥}_{F}^{2}+{∥C^{\prime }∥}_{*}\\ \; & s.t.\;C^{\prime }=\phi \left(C^{1},C^{2},\ldots ,C^{V}\right) \end{array}$$

where ${∥\cdot ∥}_{*}$ denotes the tensor nuclear norm, and ϕ(·) denotes the third-order tensor $C^{\prime }$ generated by concatenating the similarity graphs of all omics and then applying a rotation to its second and third dimensions. Then, an attention mechanism is introduced to learn the weight of each omics. First, the similarity graph of each omics is constructed as $C=\left[C^{1},C^{2},\ldots ,C^{V}\right]\in R^{N\times (N\times V)}$, where $C^{v}\in R^{N\times N}$, and the attention coefficient matrix $W\in R^{(N\times V)\times V}$ is then initialized as an all-one matrix. Finally, the leakyrelu activation function and softmax normalization are sequentially applied to the above linearly transformed results to obtain the attention weight matrix for each omics:

(8) $$M=\mathrm{softmax}\left(\mathrm{LeakyReLU}(CW)\right)$$

where $M=\left[m^{1},m^{2},\ldots ,m^{V}\right]\in R^{N\times V}$, and each column represents the weight of the corresponding omics across all samples. Accordingly, the integrated similarity matrix based on the attention mechanism is defined as:

(9) $$C_{F}=\left(m^{1}1\right)⊙C^{1}+\left(m^{2}1\right)⊙C^{2}+\cdots +\left(m^{V}1\right)⊙C^{V}$$

where $C_{F}\in R^{N\times N}$ denotes the integrated similarity graph, $1\in R^{1\times N}$ is an all-one vector, and ⊙ denotes the Hadamard product of matrices. Subsequently, we use $C_{F}$ to construct the similarity matrix $S=f_{1}\left(C_{F}\right)$, where $s_{ij}=\frac{c_{ij}+c_{ji}}{2c_{\mathrm{sum}}}$ for i≠j, and $s_{ij}=1$ for i=j. Here, $C_{\mathrm{sum}}$ is defined as the sum of all off-diagonal elements in the *i*-th row of $C_{F}$. The target distribution is then defined as $P=f_{2}\left(C_{F}\right)$, with $p_{ij}=\frac{s_{ij}^{2}}{\sum_{i}s_{ij}}/\frac{s_{ij}^{2}}{\sum_{i}s_{ij}}$. Finally, we minimize the Kullback-Leibler divergence between the target distribution *P* and the consensus similarity graph $C_{F}$:

(10) $$KL\left(P,C_{F}\right)=\sum_{i=1}^{N}\sum_{j=1}^{N}p_{i,j}\log\frac{p_{i,j}}{s_{i,j}}$$

By integrating the loss functions of all above components, the final loss function is given by:

(11) $$\begin{array}{cc} \; & \min\;\sum_{v=1}^{V}\frac{1}{2n}{∥X^{v}-\tilde{X^{v}}∥}_{F}^{2}+\alpha {∥W^{\left(1\right)}∥}_{2,1}+\lambda_{1}{∥Z^{v}-Z^{v}C^{v}∥}_{F}^{2}+\lambda_{2}{∥C^{v}∥}_{F}^{2}\\ \; & +\lambda_{3}{∥A^{v}-C^{v}∥}_{F}^{2}+{∥C^{\prime }∥}_{*}+\beta KL\left(P,C_{F}\right)\\ \; & s.t.\;C^{\prime }=\phi \left(C^{1},C^{2},\ldots ,C^{V}\right) \end{array}$$

where α, $\lambda_{1}$, $\lambda_{2}$, $\lambda_{3}$, and β are hyperparameters used to balance each loss term. In the MHSGTR algorithm, the autoencoder adopts a three-layer perceptron structure. The dimension of the input layer is consistent with the feature dimension of each omics dataset, which is used to represent the original features of different omics layers. The dimensions of the remaining two hidden layers are set to 1024 and 512, respectively, to mitigate differences in the dimensional scales of features across different omics and enhance feature representation capability. During the pre-training and training phases of the autoencoder, the learning rate is set to 0.0001 and 0.001, respectively. To investigate the effects of these parameters on model performance, we conducted a parametric sensitivity analysis and explored the optimal parameter combination using the grid search method, with detailed results provided in Supplemental Materials (Figures S1 and S2).

### 2.2. Evaluation Metrics

Two evaluation metrics were adopted to assess the performance of MHSGTR. First, the log-rank test was employed to calculate p-values in survival analysis, aiming to determine whether the cancer subtypes identified by MHSGTR exhibited statistically significant differences in survival outcomes. Second, a clinical label enrichment analysis was performed to statistically analyze patients’ clinical labels, evaluate distributional differences of clinical labels across distinct molecular subtypes, and assess the effectiveness of MHSGTR based on the number of enriched clinical labels. For each cancer type, six clinical indicators were chosen: age, gender, and four discrete clinicopathological parameters, pathological T, N, M stage and pathological stage. The chi-square test was applied for discrete clinical labels, while the Kruskal–Wallis test was used for numerical clinical labels. Since multiple clinical labels were tested simultaneously, the Benjamini-Hochberg FDR method was further applied for multiple hypothesis testing correction. Clinical indicators with an adjusted p < 0.05 were considered to be significantly associated with the identified cancer subtypes and were included in the number of significantly enriched clinical labels.

### 2.3. Dataset Processing

Multi-omics data of eight cancer types from TCGA (https://gdc.cancer.gov/, accessed on 1 October 2025) were used to evaluate the effectiveness of MHSGTR, including Adrenocortical Carcinoma (ACC), Bladder Urothelial Carcinoma (BLCA), Head and Neck Squamous Cell Carcinoma (HNSC), Kidney Renal Papillary Cell Carcinoma (KIRP), Liver Hepatocellular Carcinoma (LIHC), Lung Adenocarcinoma (LUAD), Stomach Adenocarcinoma (STAD) and Sarcoma (SARC). Each cancer dataset included mRNA expression profiles, DNA methylation profiles, miRNA expression profiles, and corresponding clinical information of enrolled patients. A unified preprocessing pipeline was applied to each cancer dataset. First, patients with incomplete or missing survival data were excluded. Subsequently, the final sample cohort was determined by selecting overlapping samples across all three omics datasets. For miRNA and mRNA data, features with missing values or with more than 30% zero expression values were filtered out. To mitigate the interference of extreme values and outliers, a $\log_{2}$ transformation was applied to the preprocessed miRNA and mRNA data. Finally, only features with non-zero variance were retained in both datasets. For DNA methylation data, the raw DNA methylation dataset contained approximately 486,427 CpG signal values. Quality control of the methylation signal matrix was performed using the R package ChAMP [26], followed by normalization of the methylation signal matrix to ensure consistent signal distribution across different samples. Given that multiple probes may map to the same gene, signal values of all duplicate probes were averaged, and methylation features with non-zero variance were retained for subsequent analysis. Detailed statistics for each dataset are summarized in Table 1.

### 2.4. Downstream Statistical Analysis

**Mutational Landscape Analysis:** The R package maftools (version 2.26.0) [27] was used to generate mutation waterfall plots to visualize the distribution of high-frequency mutated genes in each subtype. Genes with a mutation frequency greater than 5% were prioritized for visualization. Meanwhile, tumor mutational burden (TMB) was calculated to compare differences in genomic mutation levels among subtypes. Subsequently, the fraction of genome altered (FGA) was calculated based on copy number variation (CNV) data to evaluate the chromosomal instability level of different ACC subtypes.

**Differential Expression Analysis and KEGG Functional Enrichment Analysis:** The R package DESeq2 (version 1.50.2) [28] was adopted for differential expression analysis of mRNA data to identify subtype-specific upregulated and downregulated differentially expressed genes (DEGs). It should be noted that the input for DESeq2 was the raw mRNA count matrix, rather than the log2-transformed data used for model training, in order to satisfy the statistical assumptions of the DESeq2 model. Subsequently, to decipher the biological functions and pathway characteristics of DEGs, the R package clusterProfiler (version 4.18.4) [29] was used to perform Kyoto Encyclopedia of Genes and Genomes (KEGG) pathway enrichment analysis, aiming to decipher the significantly enriched biological pathways and reveal potential molecular regulatory mechanisms of each subtype.

**Immune Microenvironment Analysis:** The R package CIBERSORT (version 0.1.0) [30] was used to calculate the infiltration proportion of immune cells across subtypes, and statistical tests were performed to identify immune cell types showing significant differences among subtypes. Meanwhile, immune, stromal and estimate scores were calculated for each subtype using the ESTIMATE method [31], to reflect compositional heterogeneity within the tumor microenvironment.

## 3. Results

### 3.1. Comparison with Existing Multi-Omics Integration Methods

To verify the effectiveness of the proposed MHSGTR in the multi-omics cancer subtypes identification task, we conducted a comparative analysis between MHSGTR and nine state-of-the-art multi-omics integration methods, which can be categorized into three methodological categories: (1) four similarity graph-based methods: SNF [7], CIMLR [8], MOSD [32], and DSCC [33]; (2) three deep learning and similarity graph-based methods: MOCR [15], DSIR [34], and CSSEC [35]; and (3) two high-order relationship mining-based methods: SFHOP [16] and HyperTMO [18]. For a fair comparison, hyperparameters recommended by the original authors were adopted to achieve optimal performance for each method, and the number of clusters was set to range from 2 to 6.

As shown in Table 2, the experimental results demonstrated that MHSGTR achieved overall superior performance in survival analysis. Among the eight cancer datasets, MHSGTR yielded the highest −$\log_{10}$(*p*-value) on seven datasets. The *p*-value is derived from the log-rank test, which is used to evaluate the statistical difference in survival curves among different subtypes. As a transformed form of the *p*-value, a higher −$\log_{10}$(*p*-value) corresponds to a smaller *p*-value, indicating a more significant survival difference between subtypes. Taking the ACC dataset as an example, MHSGTR achieved the optimal result of 7.56, which was markedly superior to the second-best result of 6.42. For the KIRP dataset, although MHSGTR did not achieve the optimal $-\log_{10}$(*p*-value) (9.11, slightly lower than 9.69 obtained by DSCC), it still outperformed all other comparative methods.

Further analysis of the results presented in Table 3 revealed that the number of significant clinical labels identified by MHSGTR reached the highest or tied for the highest level across all eight cancer datasets. Statistical analysis based on the mean number of clinical labels demonstrated that the average number of enriched clinical labels obtained by MHSGTR across the eight datasets was 3, which outperformed all other comparative methods. This finding indicates that the subtypes identified by MHSGTR show more significant differences in clinical terms.

**Figure 3:**
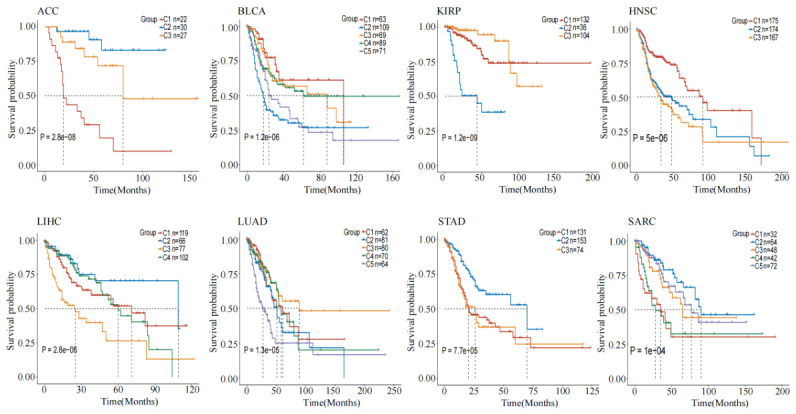
Kaplan-Meier survival curves on eight cancer datasets.

In addition, Kaplan-Meier survival curves for the eight cancer datasets are presented in Figure 3. It can be observed that the survival curves corresponding to distinct cancer subtypes exhibited a marked separation trend, which indicates that MHSGTR can effectively identify cancer subtypes with distinct prognostic characteristics.

### 3.2. Ablation Study

To further analyze the contribution of the Motif-based high-order similarity to model performance, we designed an ablation study. In this experiment, the downstream spectral clustering algorithm was kept unchanged, and adjust only the similarity matrix construction strategy, by comparing first-order similarity *S*, Motif-based similarity *M*, and hybrid similarity *A*. Here, hybrid similarity *A* corresponds to the complete similarity construction strategy in MHSGTR, namely the MHSGTR method proposed in this study. As shown in Table 4, Motif-based similarity achieved better clustering results than first-order similarity, indicating that the use of high-order proximity helps improve clustering performance. Furthermore, hybrid similarity achieved the best results across all datasets, demonstrating that first-order similarity and Motif-based high-order similarity have a certain degree of complementarity. Their integration enables the construction of a more robust sample similarity graph, thereby improving the performance of downstream spectral clustering for cancer subtype identification.

### 3.3. Analysis of Clinical Phenotypic Heterogeneity of ACC Subtypes

To further verify the effectiveness of the MHSGTR, we conducted in-depth biological analysis using ACC as an example. By integrating mRNA expression, DNA methylation and miRNA expression data, MHSGTR stratified ACC patients into three molecular subtypes with significant prognostic differences. The Kaplan–Meier survival curves in Figure 3 show significant survival stratification among the subtypes, which indicates that MHSGTR can effectively distinguish patient groups with different clinical outcomes.

**Figure 4:**
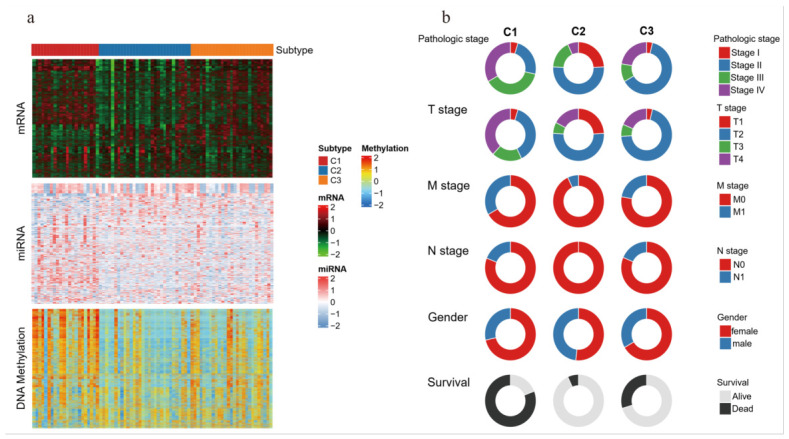
Multi-omics features and clinicopathological heterogeneity of ACC Subtypes. (**a**) Multi-omics molecular signature heatmaps showing the characteristic distribution patterns of the three subtypes. (**b**) Clinical phenotype donut charts comparing the distribution differences of the three subtypes across clinical variables.

A systematic comparison of the clinical and molecular characteristics of the three molecular subtypes was further performed. As shown in Figure 4a, each subtype exhibited relatively independent molecular characteristics at the genomic and epigenetic levels, supporting the biological rationality of the subtyping results. We then compared the heterogeneity of the molecular subtypes at the level of clinical phenotypes (Figure 4b). In terms of pathological stage, subtype C1 contained the highest proportion of stage IV patients, followed by subtype C3, while both subtype C2 and subtype C3 were predominantly composed of patients with stage II. For pathological T stage, subtype C1 showed a relatively high proportion of T4 stage patients, while subtypes C2 and C3 had a relatively high proportion of T2 stage patients. In addition, for pathological M stage, subtype C1 had the highest proportion of patients with M1 stage. For pathological N stage, all patients in subtype C2 were N0 stage, while a certain proportion of patients with N1 stage were observed in subtype C1 and subtype C3. The differences in the above clinicopathological features are consistent with the survival outcomes of each subtype to a certain extent, which helps to explain the poor clinical prognosis observed for subtype C1.

### 3.4. Genomic Heterogeneity Analysis of ACC Subtypes

**Figure 5:**
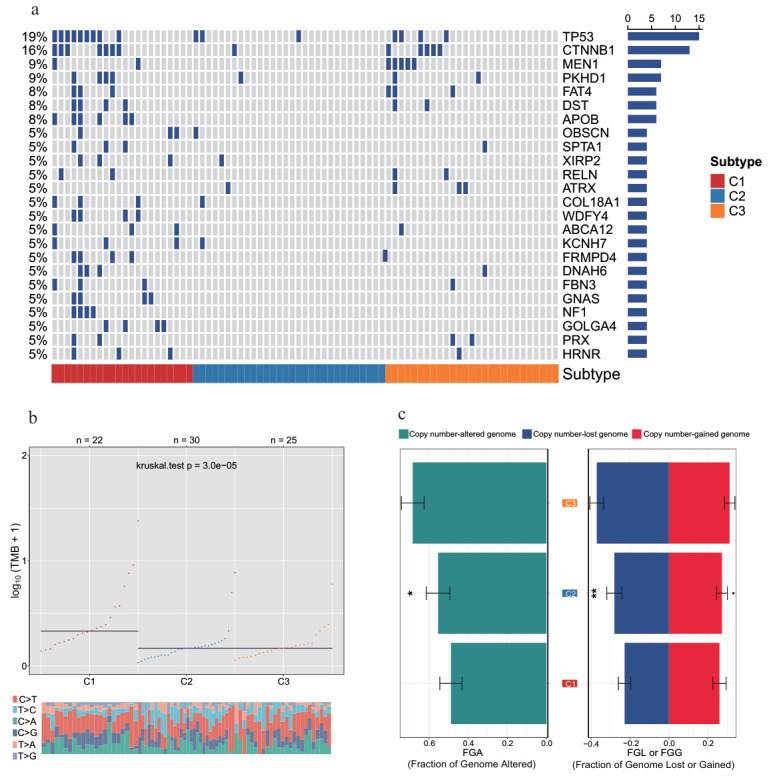
Genomic heterogeneity analysis of ACC subtypes. (**a**) Oncoplot of gene mutations in ACC subtypes. (**b**) TMB scores of ACC subtypes. (**c**) FGA scores and copy number variation proportions of ACC subtypes. * *p* < 0.05, ** *p* < 0.01.

To further decipher the genomic heterogeneity of the different molecular subtypes, we systematically compared the three ACC subtypes in terms of somatic mutation profiles, tumor mutational burden (TMB), and copy number variation (CNV). The mutation waterfall plot (Figure 5) displays the top 20 genes with the highest mutation frequency across all subtypes. The results showed that patients in C1 had a relatively higher mutation frequency, suggesting that this subtype may exhibit greater genomic instability, which could contribute to its poor prognosis. Notably, TP53 and CTNNB1 are the most common driver genes in ACC, and their aberrations have been confirmed by multiple studies to be closely associated with tumorigenesis, tumor progression, and enhanced invasiveness [36,37].

We further compared the TMB levels among different ACC subtypes. As shown in Figure 5b, the TMB level of C1 was significantly higher than that of the other two subtypes ($p=3\times 10^{-5}$), indicating that this subtype may have accumulated more somatic mutation events. In addition, as shown in Figure 5c, subtype C3 had a significantly higher fraction of genome altered (FGA) value, with more prominent copy number gain and loss events, indicating that this subtype may possess stronger chromosomal instability. These findings are consistent with the conclusions of previous studies on ACC subtypes [38].

### 3.5. Transcriptomic Heterogeneity Analysis of ACC Subtypes

**Figure 6:**
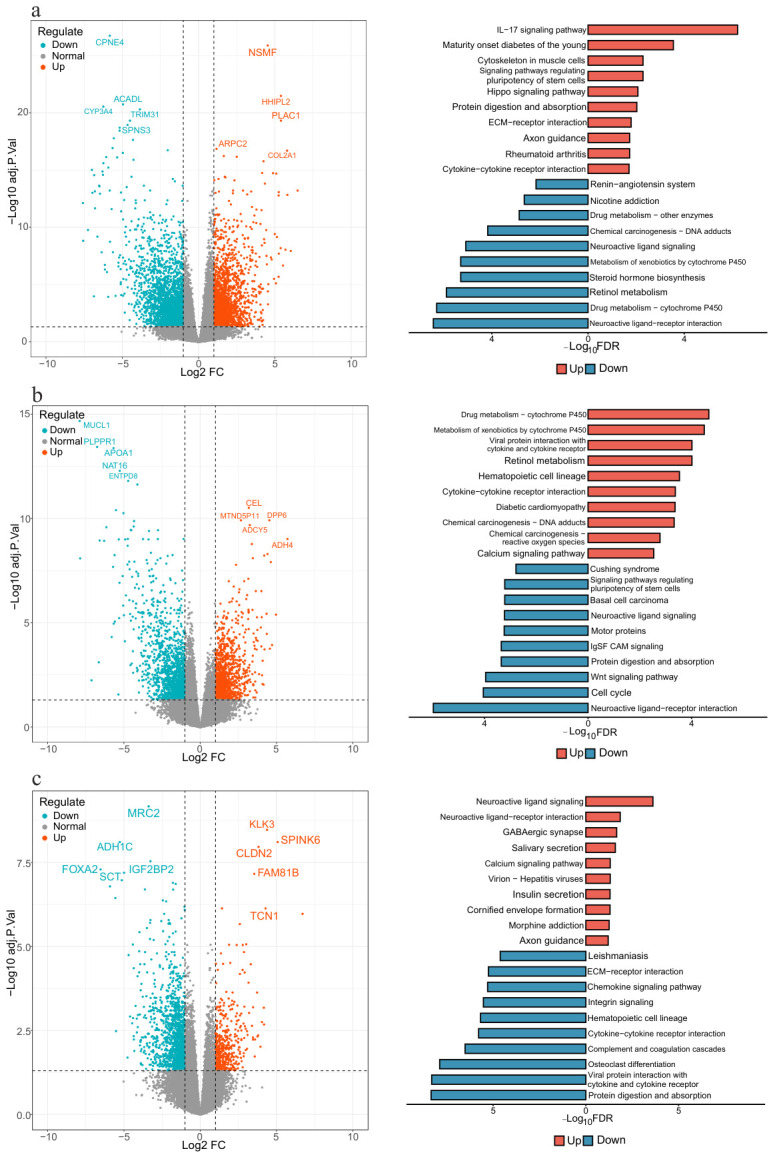
Differential genes and KEGG functional enrichment analysis of ACC subtypes. Volcano plots of differentially expressed genes and corresponding KEGG enrichment analysis results for (**a**) subtype C1, (**b**) subtype C2, and (**c**) subtype C3.

To comprehensively explore the transcriptomic signatures of different ACC molecular subtypes, we performed differential expression analysis on the mRNA expression data, with differentially expressed genes (DEGs) screened at adjusted p<0.05 and $\left|\log_{2}\left(FC\right)\right|>1$. As shown in Figure 6a, for subtype C1, a total of 1887 significantly upregulated DEGs and 2645 significantly downregulated DEGs were identified. Previous studies have confirmed that high expression of COL2A1 is closely associated with poor prognosis in ACC patients [39]. KEGG pathway enrichment analysis revealed that upregulated DEGs in C1 subtype were mainly involved in immune and inflammation-associated pathways such as the IL-17 signaling pathway, while the downregulated DEGs were significantly enriched in pathways including the neuroactive ligand-receptor interaction pathway.

As shown in Figure 6b, differential expression analysis for subtype C2 identified 1228 significantly upregulated and 1441 significantly downregulated DEGs. Previous studies have shown that aberrant expression of ADCY5 is significantly correlated with disease progression in ACC patients [40]. KEGG pathway enrichment analysis revealed that the upregulated DEGs in subtype C2 were mainly enriched in pathways such as the drug metabolism cytochrome P450 pathway, while the downregulated DEGs were significantly enriched in pathways closely related to cell proliferation, including the cell cycle pathway and the Wnt signaling pathway.

As shown in Figure 6c, subtype C3 exhibited 435 significantly upregulated and 1346 significantly downregulated DEGs. Among them, CLDN2 has been identified as a key biomarker during the progression of ACC [41]. KEGG pathway enrichment analysis demonstrated that the upregulated DEGs of subtype C3 were mainly enriched in pathways such as the neuroactive ligand signaling pathway, while the downregulated DEGs were significantly involved in immune-associated pathways, exemplified by the cytokine-cytokine receptor interaction pathway. These results demonstrate that the ACC subtypes identified by MHSGTR present significant heterogeneity in biological functions and signaling pathways, thus further supporting the biological relevance and systems-level validity of multi-omics integrative subtyping.

### 3.6. Tumor Microenvironment Characteristics of ACC Subtypes

**Figure 7:**
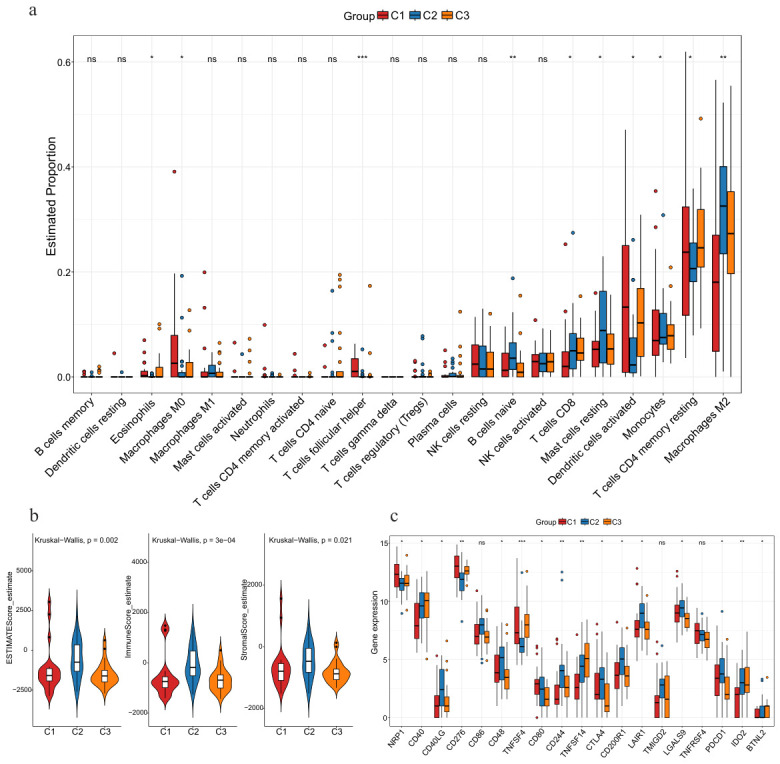
Differential analysis of immune-related characteristics across the ACC subtypes. (**a**) Infiltration levels of 22 immune cells. (**b**) TME scores were compared between subtypes. (**c**) Expression levels of immune checkpoints. * *p* < 0.05, ** *p* < 0.01, *** *p* < 0.001. ns, no significance.

To comprehensively assess the immune microenvironment properties across distinct ACC subtypes, we performed an in-depth analysis of the three subtypes in terms of immune cell infiltration levels, immune scores, stromal scores, and expression characteristics of immune-related genes. The infiltration proportions of 22 immune cell types were calculated and compared among ACC subtypes. As shown in Figure 7a, subtype C2 exhibited relatively higher infiltration levels in multiple immune cell types, such as macrophages M2, resting mast cells, and CD8+ T cells, indicating a stronger capacity for immune cell recruitment and infiltration. In contrast, subtype C1 had relatively low infiltration proportions of most immune cell types, presenting the characteristics of an immune-cold tumor. This difference may partly explain the poor prognosis of subtype C1.

Furthermore, we quantitatively evaluated the tumor microenvironment characteristics of different molecular subtypes using the ESTIMATE algorithm. As shown in Figure 7b, the three subtypes showed significant differences in immune score, stromal score, and estimate score (*p* < 0.05). Subtype C2 displayed the highest immune score and estimate score, implying a higher degree of immune cell infiltration and more abundant tumor microenvironment components in this subtype. Subtype C1 exhibited the lowest immune and stromal scores, indicating high tumor purity and an immune-cold microenvironment. This phenotype, combined with its high genomic instability and poor survival outcomes, suggests that C1 may represent a more aggressive ACC subtype with limited immune surveillance and potential resistance to immunotherapy.

The expression levels of immune checkpoints (Figure 7c) further revealed that multiple immune checkpoints (such as CD27, CD28, CD40, TNFSF4, and TNFSF14) were highly expressed in subtype C2, suggesting that this subtype may have a more active immune response capacity. In addition, several immunosuppressive checkpoint molecules, including PDCD1, LAG3, and CTLA4, were also relatively upregulated in subtype C2, suggesting a state of immune activation accompanied by enhanced immune regulation. In contrast, the expression levels of most immune-related genes were significantly downregulated in subtype C1, which is consistent with the poor prognostic characteristics of subtype C1. In summary, different molecular subtypes of ACC exhibit significant heterogeneity at the immune microenvironment level. Subtype C2 is characterized by a high degree of immune cell infiltration, elevated immune score and stromal score, and upregulation of multiple immune activation-related genes, presenting typical characteristics of an immune-hot tumor. Subtype C1 is characterized by insufficient immune cell infiltration and low immune score, which is consistent with the characteristics of an immune-cold tumor.

## 4. Conclusions and Discussion

As a highly heterogeneous and complex disease, accurate molecular subtyping of cancer represents a key scientific challenge for advancing individualized diagnosis and treatment, as well as for improving patient prognosis. Similarity graph-based multi-omics data integration methods serve as a pivotal technical approach in the field of cancer subtyping research. However, most existing methods rely on solely first-order similarity to characterize inter-sample relationships, which not only makes it difficult to fully capture the latent high-order structural information embedded in multi-omics data, but also fails to adequately leverage the complementary information across different omics modalities, thereby limiting the accuracy of subtype identification. To address these limitations, we propose a novel multi-omics data fusion framework MHSGTR based on high-order relationship mining. This framework enables mutual learning between Motif high-order similarity graphs and first-order similarity graphs to fully integrate low-order and high-order association information between samples, and subsequently fully exploits complementary information across different omics via third-order tensor-based regularization and attention mechanism-guided graph fusion, thus enhancing the accuracy of cancer subtyping. Comparative experiments on eight TCGA cancer datasets, along with a detailed case study of ACC, demonstrate that the MHSGTR algorithm can effectively integrate multi-omics information to achieve superior clustering performance, and identifies cancer subtypes with statistically significant biological differences.

The main contributions of the proposed MHSGTR method can be summarized as follows. First, we design a Motif topology-driven high-order similarity learning strategy to fully explore the latent high-order neighborhood relationships among samples. This strategy enhances the structural representation capability of the similarity graph in each omics view, thereby improving the inter-class separability of the similarity graph structure. Second, we capture high-order interactive information across omics data via third-order tensor-based regularization, and integrate an attention-based adaptive fusion module to dynamically assign weights to different omics data, thereby generating the final consensus similarity graph.

In the case analysis of ACC, the MHSGTR method stratified ACC patients into three molecular subtypes with statistically significant prognostic differences. At the clinical phenotypic level, the ACC subtypes exhibited clear clinical heterogeneity. Subtype C1 showed high invasiveness and advanced pathological features, providing a clinical basis for its poor prognosis. At the genomic level, subtype C1 displayed a significantly higher mutation frequency and TMB level, while subtype C3 had a significantly higher FGA value and more pronounced chromosomal instability. These findings are highly consistent with the conclusions of previous studies on ACC molecular subtypes [38]. In the differential expression analysis and enrichment analysis of transcriptional profiles, the three ACC subtypes exhibited distinct gene expression patterns and pathway enrichment signatures, indicating that the ACC molecular subtypes identified by MHSGTR have significant heterogeneity in biological functions and signaling pathways. In the analysis of the tumor immune microenvironment, significant differences were observed among different subtypes in terms of immune cell infiltration patterns, immune score, and immune checkpoint expression. Among them, subtype C2 exhibited the characteristics of an immune-hot tumor, with high levels of immune cell infiltration, elevated immune-related scores, and upregulation expression of immune checkpoint molecules. In contrast, subtype C1 displayed an immune-cold tumor phenotype with insufficient immune cell infiltration and suppressed immune activity, which is highly consistent with the poor clinical prognosis of this subtype. Together, these results support the biological relevance of MHSGTR in cancer subtype identification.

Although this study has achieved promising results, several limitations should be acknowledged. First, this study mainly integrates three types of omics data, namely mRNA expression, miRNA expression, and DNA methylation, for cancer subtype identification. However, tumor heterogeneity is a complex biological phenomenon jointly driven by multi-level molecular regulation. In addition to the above-mentioned omics data, proteomics, metabolomics, spatial transcriptomics, and single-cell omics may also provide important complementary information. Therefore, using only these three types of omics data may not fully characterize all aspects of tumor heterogeneity. Notably, the MHSGTR framework is not dependent on any specific type of omics data; instead, it models the topological relationships among samples from the perspective of graph structure. Thus, it has the potential to be extended to other omics modalities. In future work, we will further explore the applicability of MHSGTR to more types of omics data, so as to more comprehensively elucidate the molecular mechanisms underlying cancer subtypes. Second, in the exploration of high-order relationships among samples, we introduced the Motif structure to enhance the representation ability of similarity graphs. However, in complex network science, high-order relationships can be represented in diverse forms, and distinct connection patterns of nodes usually correspond to different functional modules. Future work may attempt to introduce more diverse representations of high-order structures into the model, so as to further improve the quality of similarity graph construction. Finally, because datasets with matched mRNA, miRNA, DNA methylation, survival information, and complete clinical labels are relatively limited, this study has not yet validated the robustness of the molecular subtypes identified by MHSGTR in independent external cohorts, which constitutes a limitation of our work. In future research, we will perform external cohort validation to comprehensively assess the reproducibility of the MHSGTR method for cancer subtyping.

In summary, the proposed MHSGTR clustering algorithm enables effective integration of multi-omics data and provides a reliable technical approach for cancer subtypes identification. By exploring the high-order topological structure among samples, MHSGTR strengthens the discriminative capability of similarity graphs for individual samples, thereby effectively improving the accuracy of clustering results. This study provides novel research insights and methodological support for multi-omics data integration and high-order relationship modeling, and also offers a referable statistical analysis framework for precise cancer subtyping.

## Figures and Tables

**Table 1:** The details of the eight cancer datasets utilized in this study.

Dataset	mRNA	DNA Methylation	miRNA	Sample Number
ACC	27,800	13,307	579	79
BLCA	20,419	11,988	617	401
KIRP	30,439	18,597	544	272
HNSC	29,835	18,402	607	516
LIHC	26,778	18,133	577	364
LUAD	32,720	18,110	595	357
STAD	37,469	18,212	541	358
SARC	29,170	18,150	501	258

**Table 2:** −$\log_{10}$(*p*-value)/number of clusters of MHSGTR and comparative methods.

Method	ACC	BLCA	KIRP	HNSC	LIHC	LUAD	STAD	SARC
SNF	3.70/3	3.00/4	4.18/5	3.50/4	2.37/3	2.84/5	1.04/4	2.25/3
CIMLR	3.22/6	2.78/3	3.10/3	2.76/4	2.49/5	1.53/5	2.41/5	1.41/4
MOSD	4.12/4	2.54/4	5.15/3	3.07/3	2.54/4	2.32/3	2.03/4	2.69/3
DSCC	6.30/3	4.00/3	9.69/6	3.32/5	2.69/4	2.70/4	2.43/5	3.52/5
MOCR	4.83/3	4.28/4	5.43/5	3.26/3	3.72/5	3.45/3	2.87/6	3.25/2
DSIR	5.19/4	3.61/4	4.37/3	3.38/4	3.20/3	2.47/3	2.89/3	3.54/4
CSSEC	5.37/3	4.29/2	5.35/2	3.14/3	3.94/2	3.35/2	2.90/3	2.28/5
SFHOP	5.40/3	4.12/5	5.26/3	5.05/5	3.34/4	2.56/4	3.06/4	3.46/3
HyperTMO	6.42/3	4.90/4	6.34/4	4.54/3	4.97/3	3.29/6	3.62/5	3.58/3
MHSGTR	7.56/3	5.86/5	9.11/3	5.31/3	5.57/4	4.83/5	4.11/3	4.02/5

**Table 3:** Number of significant clinical labels for MHSGTR and comparative methods.

Method	ACC	BLCA	KIRP	HNSC	LIHC	LUAD	STAD	SARC
SNF	2	2	3	2	2	1	0	2
CIMLR	2	3	2	2	1	1	1	0
MOSD	2	2	4	1	3	2	1	1
DSCC	2	3	4	3	3	2	1	1
MOCR	2	2	4	2	2	2	1	2
DSIR	3	2	3	3	2	1	2	2
CSSEC	3	3	4	2	2	2	1	1
SFHOP	2	3	2	3	2	1	2	2
HyperTMO	2	3	3	2	3	2	2	1
MHSGTR	3	4	5	3	3	2	2	2

**Table 4:** −$\log_{10}$(*p*-value) of different similarity construction strategies on eight cancer datasets.

Dataset	First-Order Similarity	Motif-Based Similarity	Hybrid Similarity
ACC	5.07	5.92	7.56
BLCA	3.55	4.17	5.86
KIRP	6.48	7.82	9.11
HNSC	3.82	4.56	5.31
LIHC	3.61	4.06	5.57
LUAD	2.79	3.54	4.83
STAD	2.64	3.19	4.11
SARC	2.59	3.47	4.02

## Data Availability

The TCGA multi-omics datasets used in this study are publicly available at https://gdc.cancer.gov/ (accessed on 1 October 2025).

## Supplementary Materials

The following supporting information can be downloaded at: https://www.mdpi.com/article/10.3390/genes17050587/s1, Figure S1. Sensitivity analysis of parameters $\lambda_{1}$ and $\lambda_{2}$ in MHSGTR; Figure S2. Sensitivity analysis of parameters $\lambda_{3}$ in MHSGTR.
