# Supplementary material for: Multi-Omics Data Integration Clustering for Cancer Subtypes Identification Based on Motif High-Order Similarity Graph and Tensor Regularization
Source: Genes (Basel). 2026 May 21;17(5):587. doi: 10.3390/genes17050587 (PMC13205267; doi:10.3390/genes17050587)
Supplement: Supplementary file 1 [file genes-17-00587-s001.zip › genes-4305021-supplementary.pdf]

Article

Supplemental materials for "Multi-Omics Data Integration Clustering for Cancer Subtypes Identification Based on Motif High-Order Similarity Graph and Tensor Regularization"

Hongbin Yan and Fuyan Hu *

School of Mathematics and Statistics, Wuhan University of Technology, Wuhan 430070, China; 347507@whut.edu.cn
* Correspondence: fuyanhu@whut.edu.cn

1. Sensitivity analysis of parameters in MHSGTR

There are five parameters in the MHSGTR algorithm: α, λ_1, λ_2, λ_3, and β. Among them, α and β were set with reference to the parameter configurations reported by Zhang et al. [1] and Zheng et al. [2], respectively. we adopt a grid search strategy to determine the optimal combination of λ_1, λ_2, and λ_3. First, we search for the optimal values of λ_1 and λ_2 from the set $\{10^{-3}, 10^{-2}, 10^{-1}, 1, 10^1, 10^2, 10^3\}$. Then, with λ_1, λ_2 fixed, we search for the optimal value of λ_3 within the same interval. Figure S1 shows the variation in $-\log_{10}(p\text{-}value)$ corresponding to different values of λ_1 and λ_2. It can be seen that MHSGTR exhibits relatively stable performance to a certain extent. However, compared with λ_1, the model shows a certain degree of sensitivity to λ_2. When λ_2 is in the range $[0.1, 10]$, the model tends to yield a better $-\log_{10}(p\text{-}value)$.

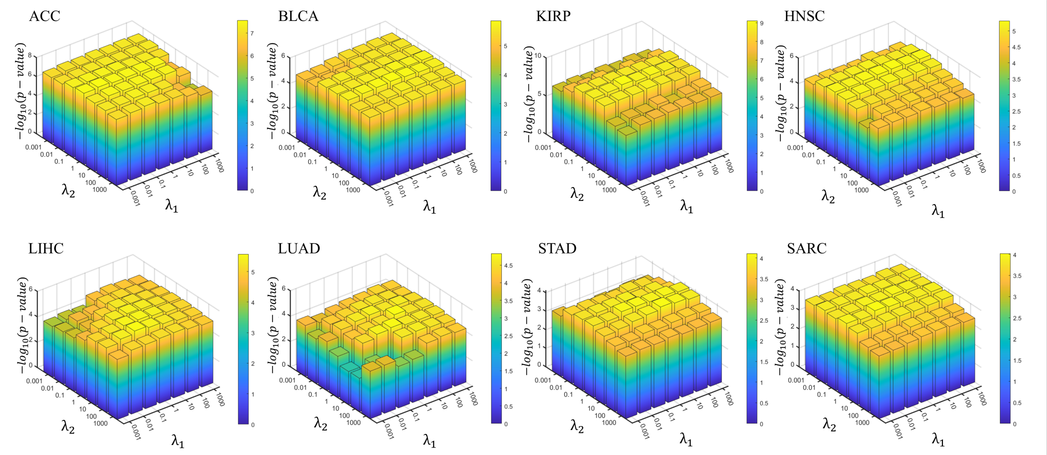

Figure S1. Sensitivity analysis of parameters λ_1 and λ_2.

With λ_1 and λ_2 fixed, we then search for the optimal value of λ_3 within the set $\{10^{-3}, 10^{-2}, 10^{-1}, 1, 10^1, 10^2, 10^3\}$. As shown in Figure S2, the overall performance of MHSGTR presents a trend of first increasing and then decreasing. As λ_3 increases, the model assigns higher weights to the Motif-based high-order similarity learning loss term, thereby enabling more adequate mining of high-order association information between samples and improving the model's ability to capture complex topological patterns. However, when λ_3 increases to a relatively high level, the model will overemphasize local high-order structural information, which may cause it to ignore global structural information and

complementary features across different omics layers, ultimately leading to a decline in clustering performance.

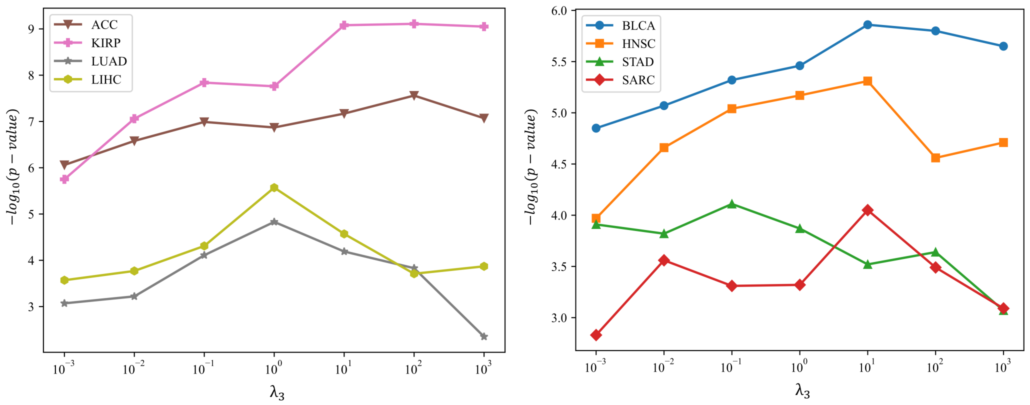

Figure S2. Sensitivity analysis of parameters λ_3.

References

1. Zhang, Y.; Lu, Z.; Wang, S. Unsupervised feature selection via transformed auto-encoder. *Knowledge-Based Systems* **2021**, *215*, 106748.
2. Zheng, Y.; Huang, H.; Luo, Y.; et al. Multi-view Subspace Tensorization with Attentive Clustering Embedding. *Neural Networks* **2025**, *196*, 108344.

Disclaimer/Publisher's Note: The statements, opinions and data contained in all publications are solely those of the individual author(s) and contributor(s) and not of MDPI and/or the editor(s). MDPI and/or the editor(s) disclaim responsibility for any injury to people or property resulting from any ideas, methods, instructions or products referred to in the content.
